# Supplementary figures and images for: TNFα-induced metabolic reprogramming drives an intrinsic anti-viral state
Source: PLoS Pathog. 2022 Jul 14;18(7):e1010722. doi: 10.1371/journal.ppat.1010722 (PMC9321404; doi:10.1371/journal.ppat.1010722)

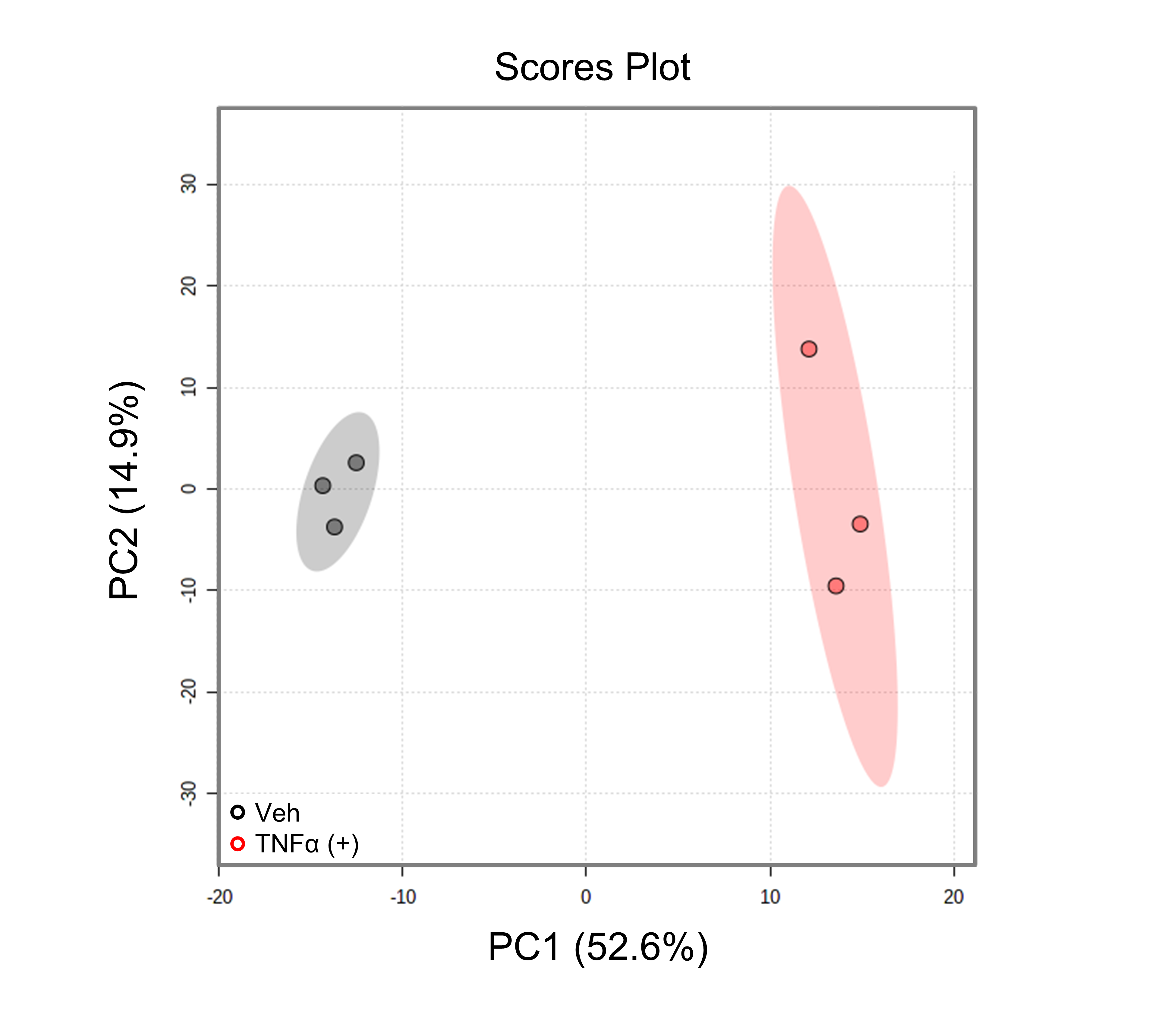

Supplement: S1 Fig — Cells were harvested for proteomics analysis (S5 Table). PCA of a subset of proteins from proteomics analysis involved in metabolism; analysis described in materials and methods (S6 Table). Data shared with Fig 4A. (TIF) [file ppat.1010722.s001.TIF]

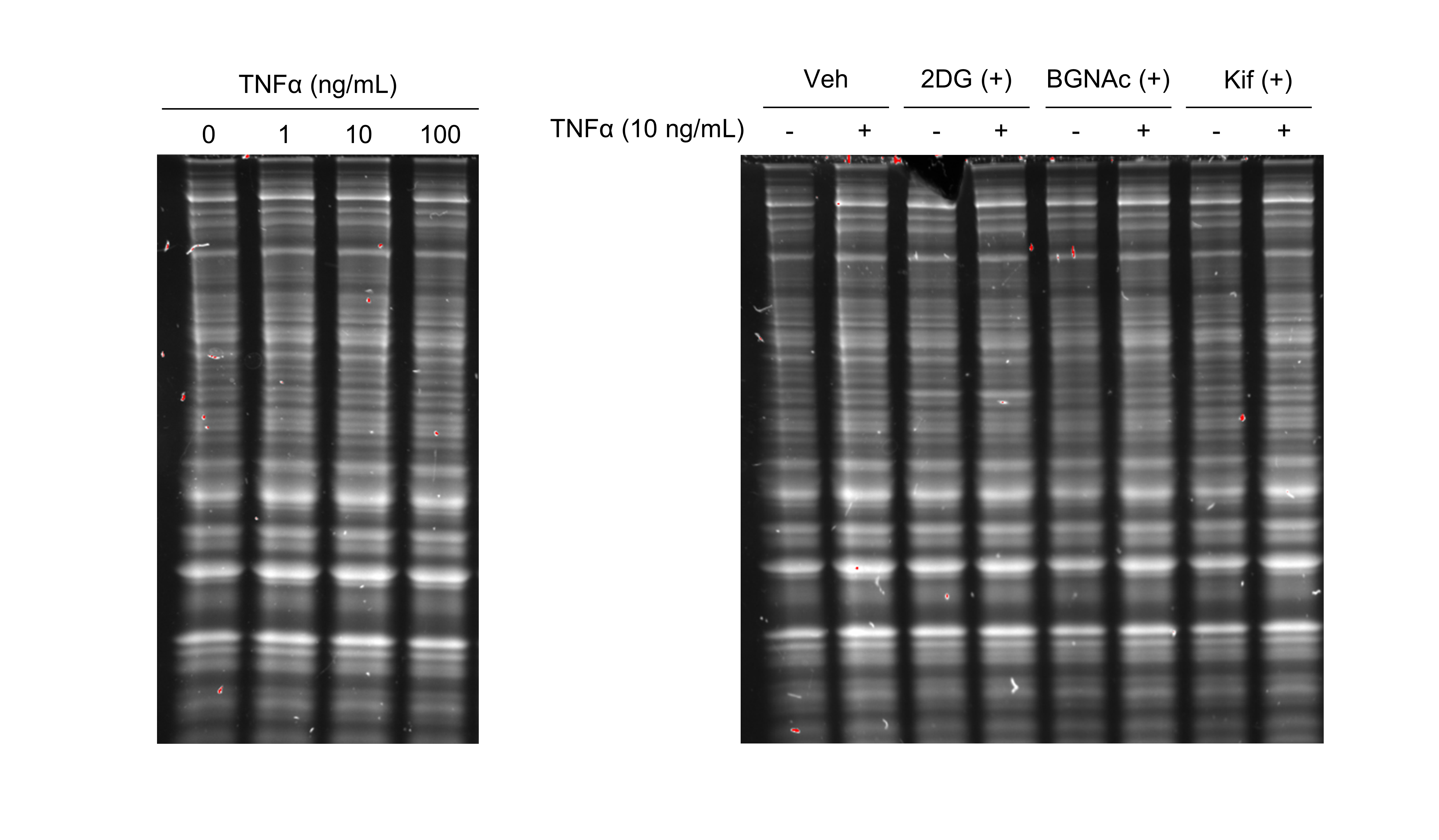

Supplement: S2 Fig — (TIF) [file ppat.1010722.s002.TIF]

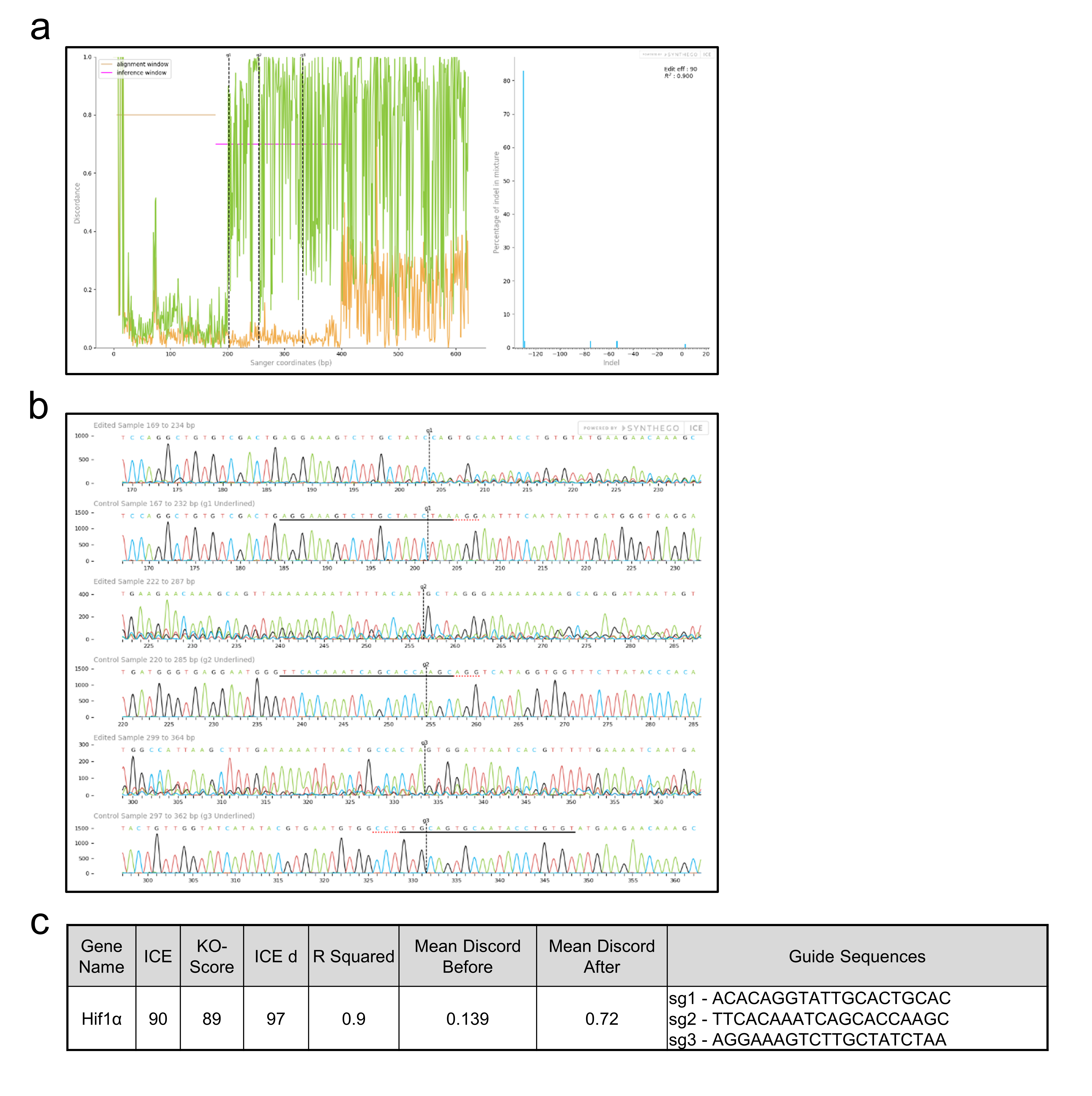

Supplement: S3 Fig — HFFs treated with CRISPR Cas9-RNP containing guides for HIF1A or a non-target guide (ntg) to generate knockout (KO) cell lines. HIF1A and ntg targeted cells harvested for genomic DNA and HIF1A gene locus amplified with sequencing primers. Sanger sequencing results uploaded to Synthego’s ICE tool. a Alignment plot, left, showing control (orange) and edited (green) sequences. Vertical dotted lines indicate guide sequences in relation to Sanger sequence coordinates. Indel plot, right, showing the predicted range of insertions and deletions in the edited gene locus. b Trace files of HIF1A (edited sample) and ntg (control sample) targeted cells spanning the cut site of HIF1A gene locus targeted by sgRNAs. Guide sequences underlined by black solid line in the control trace, PAM sequences denoted by dotted red underline and vertical dotted lines indicate expected cut site. c Table from ICE analysis displaying ICE and KO score as well as sgRNA sequences towards HIF1A. (TIF) [file ppat.1010722.s003.TIF]

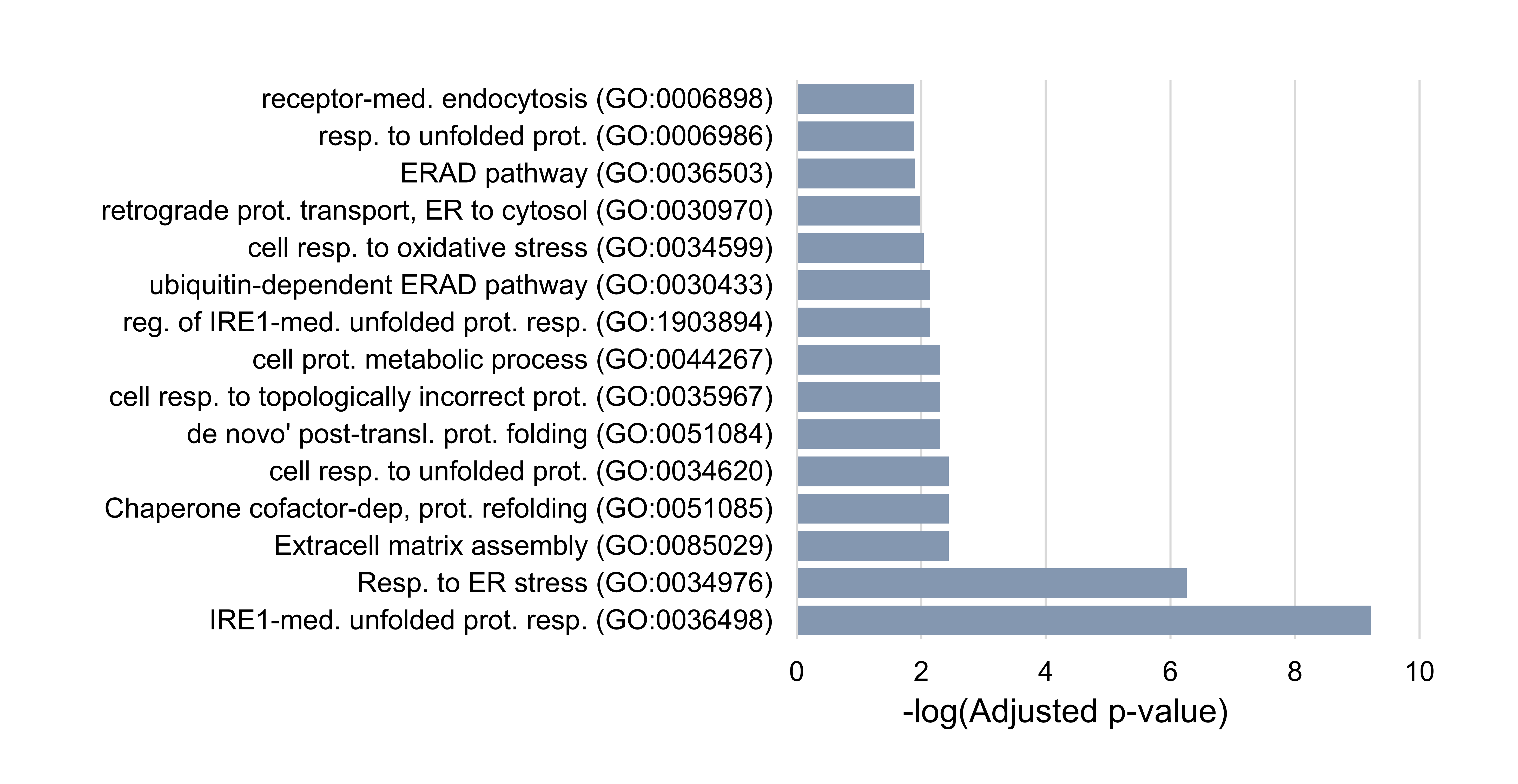

Supplement: S4 Fig — Cells harvested and analyzed for protein abundance (S5 Table). Ontology analysis of TNFα-induced proteins that were significantly more abundant upon co-treatment with 2DG. Bar graph represents the FDR-values from the top 15 GO-terms (S8 Table). (TIF) [file ppat.1010722.s004.TIF]

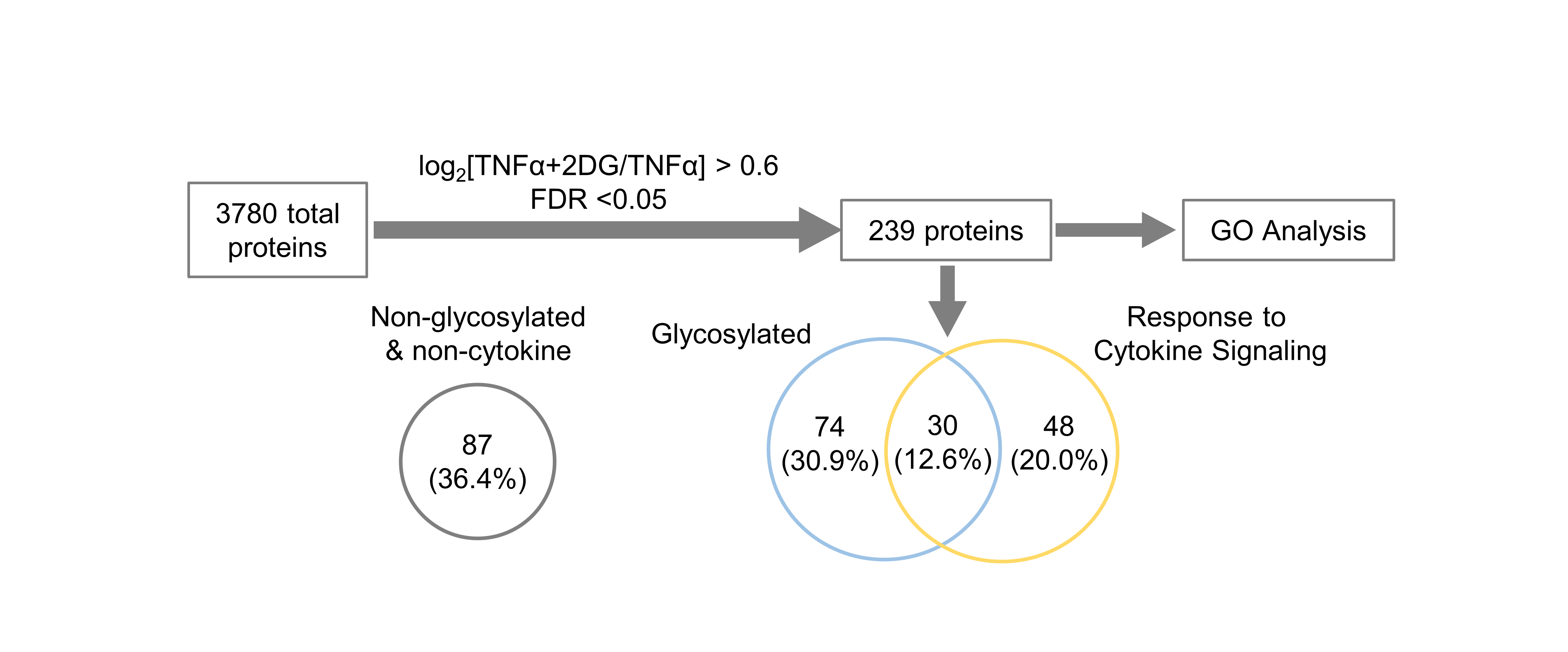

Supplement: S5 Fig — Cells harvested and analyzed for protein abundance (S5 Table). Statistical parameters applied to proteomics data, described in materials and methods, to generate a list of proteins significantly induced by TNFα treatment but depleted in cells co-treated with TNFα and 2DG. Protein list submitted for Gene Ontology (GO) analysis (Fig 6B) and scanned against databases of known glycosylated proteins (described in materials and methods) or ‘Response to Cytokine Signaling’ [GO:0034097] (S7 Table). Venn Diagram represents overlap of proteins involved in cytokine signaling (yellow) and glycosylated (blue). (TIF) [file ppat.1010722.s005.TIF]

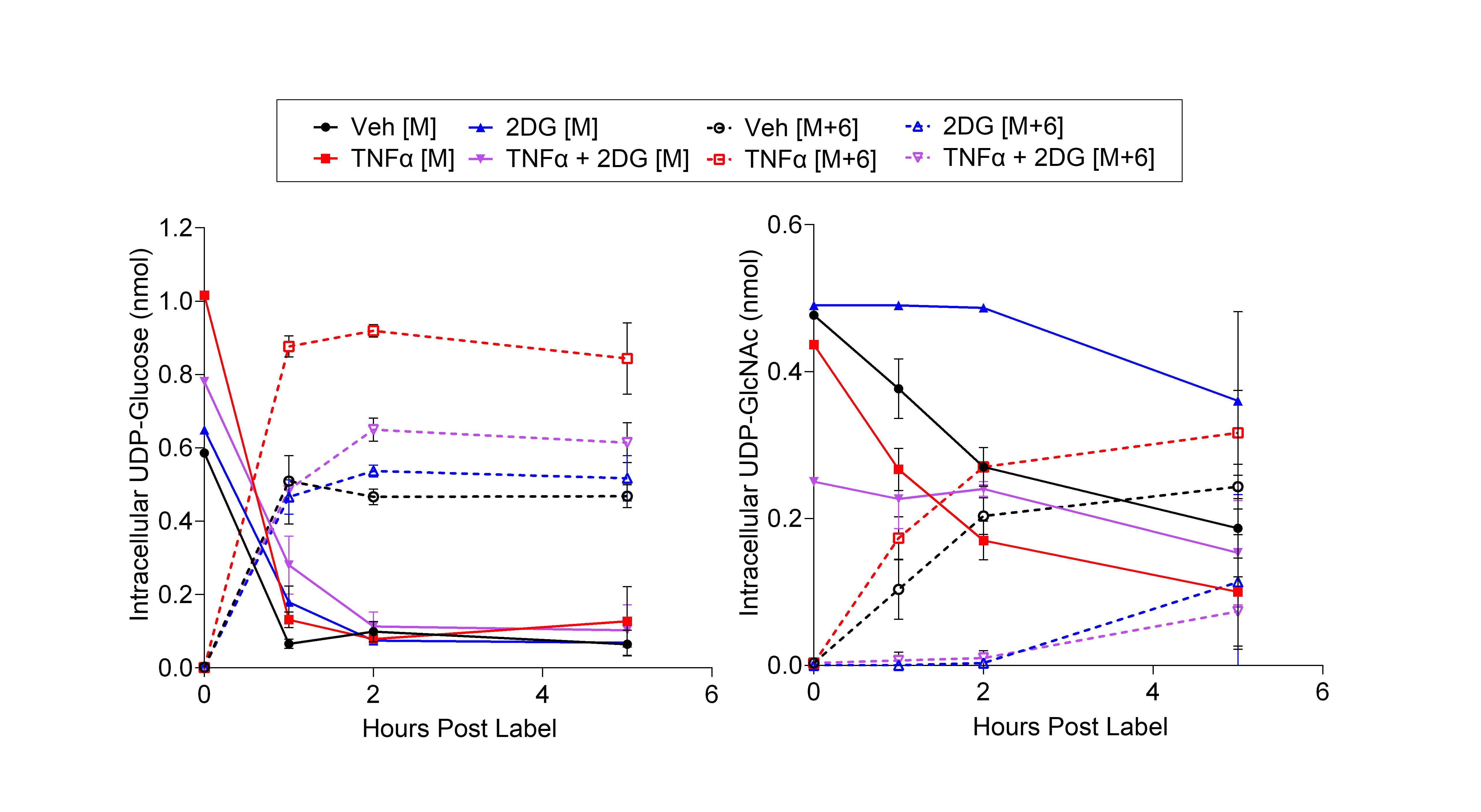

Supplement: S6 Fig — Cellular extracts were harvested at t = 0, 1, 2 and 5 hr post-label addition. UDP-Glc or UDP-GlcNAc intracellular isotopologue abundances were quantified by LC-MS/MS (mean ± SD, n = 3). Solid lines represent intracellular abundances of unlabeled 12C metabolite species, dashed lines represent intracellular abundances of 13C-labeled metabolite species. M refers to the 12C unlabeled species and M+n represents detection of a 13C-labeled species where n represents the number of additional mass units detected by mass spec. (TIF) [file ppat.1010722.s006.TIF]
