# Supplementary material for: TNFα-induced metabolic reprogramming drives an intrinsic anti-viral state
Source: PLoS Pathog. 2022 Jul 14;18(7):e1010722. doi: 10.1371/journal.ppat.1010722 (PMC9321404; doi:10.1371/journal.ppat.1010722)
Supplement: S1 Data — (DOCX) [file ppat.1010722.s015.docx]

**R-history for Fig. 1/Supplementary Table 1 & 2**

# PID of current job: 38840

mSet<-InitDataObjects("conc", "stat", FALSE)

mSet<-Read.TextData(mSet, "Replacing_with_your_file_path", "rowu", "disc");

mSet<-SanityCheckData(mSet)

mSet<-ReplaceMin(mSet);

mSet<-PreparePrenormData(mSet)

mSet<-Normalization(mSet, "NULL", "LogNorm", "AutoNorm", ratio=FALSE, ratioNum=20)

mSet<-PlotNormSummary(mSet, "norm_0_", "png", 72, width=NA)

mSet<-PlotSampleNormSummary(mSet, "snorm_0_", "png", 72, width=NA)

mSet<-Volcano.Anal(mSet, FALSE, 2.0, 0, F, 0.1, TRUE, "raw")

mSet<-PlotVolcano(mSet, "volcano_0_",1, "png", 72, width=NA)

mSet<-Volcano.Anal(mSet, FALSE, 1.5, 0, F, 0.05, TRUE, "fdr")

mSet<-PlotVolcano(mSet, "volcano_1_",1, "png", 72, width=NA)

mSet<-Volcano.Anal(mSet, FALSE, 1.5, 0, F, 0.05, TRUE, "fdr")

mSet<-PlotVolcano(mSet, "volcano_2_",1, "png", 72, width=NA)

mSet<-Volcano.Anal(mSet, FALSE, 1.0, 0, F, 0.99999, TRUE, "fdr")

mSet<-PlotVolcano(mSet, "volcano_3_",1, "png", 72, width=NA)

mSet<-PlotHeatMap(mSet, "heatmap_0_", "png", 72, width=NA, "norm", "row", "euclidean", "ward.D","bwm", "overview", T, T, NULL, T, F)

mSet<-GetGroupNames(mSet, "null")

colVec<-c("#ff0000","#000000")

shapeVec<-c(0,0)

mSet<-UpdateGraphSettings(mSet, colVec, shapeVec)

mSet<-PlotHeatMap(mSet, "heatmap_1_", "png", 72, width=NA, "norm", "row", "euclidean", "ward.D","bwm", "overview", T, T, NULL, T, F)

mSet<-PCA.Anal(mSet)

mSet<-PlotPCAPairSummary(mSet, "pca_pair_0_", "png", 72, width=NA, 5)

mSet<-PlotPCAScree(mSet, "pca_scree_0_", "png", 72, width=NA, 5)

mSet<-PlotPCA2DScore(mSet, "pca_score2d_0_", "png", 72, width=NA, 1,2,0.95,0,0)

mSet<-PlotPCALoading(mSet, "pca_loading_0_", "png", 72, width=NA, 1,2);

mSet<-PlotPCABiplot(mSet, "pca_biplot_0_", "png", 72, width=NA, 1,2)

mSet<-PlotPCA3DLoading(mSet, "pca_loading3d_0_", "json", 1,2,3)

**R-history for Fig. 3/Supplementary Table 3**

# PID of current job: 13813

mSet<-InitDataObjects("conc", "stat", FALSE)

mSet<-Read.TextData(mSet, "Replacing_with_your_file_path", "rowu", "disc");

mSet<-SanityCheckData(mSet)

mSet<-ReplaceMin(mSet);

mSet<-PreparePrenormData(mSet)

mSet<-Normalization(mSet, "NULL", "LogNorm", "AutoNorm", ratio=FALSE, ratioNum=20)

mSet<-PlotNormSummary(mSet, "norm_0_", "png", 72, width=NA)

mSet<-PlotSampleNormSummary(mSet, "snorm_0_", "png", 72, width=NA)

mSet<-ANOVA.Anal(mSet, F, 0.05, "fisher", FALSE)

mSet<-PlotANOVA(mSet, "aov_0_", "png", 72, width=NA)

mSet<-ANOVA.Anal(mSet, F, 0.05, "tukey", FALSE)

mSet<-PlotANOVA(mSet, "aov_1_", "png", 72, width=NA)

mSet<-ANOVA.Anal(mSet, F, 0.99999, "tukey", FALSE)

mSet<-PlotANOVA(mSet, "aov_2_", "png", 72, width=NA)

mSet<-PCA.Anal(mSet)

mSet<-PlotPCAPairSummary(mSet, "pca_pair_0_", "png", 72, width=NA, 5)

mSet<-PlotPCAScree(mSet, "pca_scree_0_", "png", 72, width=NA, 5)

mSet<-PlotPCA2DScore(mSet, "pca_score2d_0_", "png", 72, width=NA, 1,2,0.95,0,0)

mSet<-PlotPCALoading(mSet, "pca_loading_0_", "png", 72, width=NA, 1,2);

mSet<-PlotPCABiplot(mSet, "pca_biplot_0_", "png", 72, width=NA, 1,2)

mSet<-PlotPCA3DLoading(mSet, "pca_loading3d_0_", "json", 1,2,3)

mSet<-GetGroupNames(mSet, "null")

colVec<-c("#004dff","#ff0303","#d000ff","#000000")

shapeVec<-c(0,0,0,0)

mSet<-UpdateGraphSettings(mSet, colVec, shapeVec)

mSet<-PlotPCA2DScore(mSet, "pca_score2d_1_", "png", 72, width=NA, 1,2,0.95,0,0)

mSet<-PlotHeatMap(mSet, "heatmap_0_", "png", 72, width=NA, "norm", "row", "euclidean", "ward.D","bwm", "overview", T, T, NULL, T, F)

**R-history for Fig. 4a/Supplementary Table 6**

# PID of current job: 3115850

mSet<-InitDataObjects("conc", "stat", FALSE)

mSet<-Read.TextData(mSet, "Replacing_with_your_file_path", "rowu", "disc");

mSet<-SanityCheckData(mSet)

mSet<-ContainMissing(mSet)

mSet<-ContainsMetaDataFile(mSet)

mSet<-ReplaceMin(mSet);

mSet<-SanityCheckData(mSet)

mSet<-ContainMissing(mSet)

mSet<-ContainsMetaDataFile(mSet)

mSet<-FilterVariable(mSet, "iqr", "F", 25)

mSet<-PreparePrenormData(mSet)

mSet<-Normalization(mSet, "NULL", "NULL", "AutoNorm", ratio=FALSE, ratioNum=20)

mSet<-PlotNormSummary(mSet, "norm_0_", "png", 72, width=NA)

mSet<-PlotSampleNormSummary(mSet, "snorm_0_", "png", 72, width=NA)

mSet<-PlotHeatMap(mSet, "heatmap_0_", "png", 72, width=NA, "norm", "row", "euclidean", "ward.D","bwm", "overview", T, T, NULL, T, F)

mSet<-GetGroupNames(mSet, "null")

colVec<-c("#000000","#ff0000")

shapeVec<-c(0,0)

mSet<-UpdateGraphSettings(mSet, colVec, shapeVec)

mSet<-PlotHeatMap(mSet, "heatmap_1_", "png", 72, width=NA, "norm", "row", "euclidean", "ward.D","bwm", "overview", T, T, NULL, T, F)

mSet<-Volcano.Anal(mSet, FALSE, 2.0, 0, F, 0.1, TRUE, "raw")

mSet<-PlotVolcano(mSet, "volcano_0_",1, "png", 72, width=NA)

mSet<-Volcano.Anal(mSet, FALSE, 1.5, 1, F, 0.1, TRUE, "fdr")

mSet<-PlotVolcano(mSet, "volcano_1_",1, "png", 72, width=NA)

mSet<-UpdateLoadingCmpd(mSet, "GCLM")

mSet<-PlotCmpdView(mSet, "GCLM", "png", 72, width=NA)

mSet<-UpdateLoadingCmpd(mSet, "FTH1")

mSet<-PlotCmpdView(mSet, "FTH1", "png", 72, width=NA)

mSet<-UpdateLoadingCmpd(mSet, "EXTL2")

mSet<-PlotCmpdView(mSet, "EXTL2", "png", 72, width=NA)

mSet<-UpdateLoadingCmpd(mSet, "SLC39A14")

mSet<-PlotCmpdView(mSet, "SLC39A14", "png", 72, width=NA)

mSet<-UpdateLoadingCmpd(mSet, "AMPD3")

mSet<-PlotCmpdView(mSet, "AMPD3", "png", 72, width=NA)

mSet<-PCA.Anal(mSet)

mSet<-PlotPCAPairSummary(mSet, "pca_pair_0_", "png", 72, width=NA, 5)

mSet<-PlotPCAScree(mSet, "pca_scree_0_", "png", 72, width=NA, 5)

mSet<-PlotPCA2DScore(mSet, "pca_score2d_0_", "png", 72, width=NA, 1,2,0.95,0,0)

mSet<-PlotPCALoading(mSet, "pca_loading_0_", "png", 72, width=NA, 1,2);

mSet<-PlotPCABiplot(mSet, "pca_biplot_0_", "png", 72, width=NA, 1,2)

mSet<-PlotPCA3DLoading(mSet, "pca_loading3d_0_", "json", 1,2,3)

**R-history for Fig. 5a/Supplementary Table 5**

# PID of current job: 2874974

mSet<-InitDataObjects("conc", "stat", FALSE)

mSet<-Read.TextData(mSet, "Replacing_with_your_file_path", "rowu", "disc");

mSet<-SanityCheckData(mSet)

mSet<-ReplaceMin(mSet);

mSet<-SanityCheckData(mSet)

mSet<-FilterVariable(mSet, "none", "F", 25)

mSet<-PreparePrenormData(mSet)

mSet<-Normalization(mSet, "NULL", "NULL", "AutoNorm", ratio=FALSE, ratioNum=20)

mSet<-PlotNormSummary(mSet, "norm_0_", "png", 72, width=NA)

mSet<-PlotSampleNormSummary(mSet, "snorm_0_", "png", 72, width=NA)

mSet<-PCA.Anal(mSet)

mSet<-PlotPCAPairSummary(mSet, "pca_pair_0_", "png", 72, width=NA, 5)

mSet<-PlotPCAScree(mSet, "pca_scree_0_", "png", 72, width=NA, 5)

mSet<-PlotPCA2DScore(mSet, "pca_score2d_0_", "png", 72, width=NA, 1,2,0.95,0,0)

mSet<-PlotPCALoading(mSet, "pca_loading_0_", "png", 72, width=NA, 1,2);

mSet<-PlotPCABiplot(mSet, "pca_biplot_0_", "png", 72, width=NA, 1,2)

mSet<-PlotPCA3DLoading(mSet, "pca_loading3d_0_", "json", 1,2,3)

mSet<-GetGroupNames(mSet, "null")

colVec<-c("#2600ff","#ff0000","#c86ce6","#NA")

shapeVec<-c(0,0,0,0)

mSet<-UpdateGraphSettings(mSet, colVec, shapeVec)

colVec<-c("#2600ff","#ff0000","#c86ce6","#000000")

shapeVec<-c(0,0,0,0)

mSet<-UpdateGraphSettings(mSet, colVec, shapeVec)

mSet<-PlotPCA2DScore(mSet, "pca_score2d_1_", "png", 72, width=NA, 1,2,0.95,0,0)

colVec<-c("#1c49fc","#ff0000","#c86ce6","#000000")

shapeVec<-c(0,0,0,0)

mSet<-UpdateGraphSettings(mSet, colVec, shapeVec)

mSet<-PlotPCA2DScore(mSet, "pca_score2d_2_", "png", 72, width=NA, 1,2,0.95,0,0)
